# Survival analysis of the unsafe behaviors leading to urban expressway crashes

**DOI:** 10.1371/journal.pone.0267559

**Published:** 2022-08-26

**Authors:** Ning Huajing, Yunyan Yu, Lu Bai

**Affiliations:** 1 College of Civil Engineering, Lanzhou Jiaotong University, Lanzhou, China; 2 School of Urban Construction and Transportation, Hefei University, Hefei, China; 3 Jiangsu Key Laboratory of Urban ITS, Southeast University, Jiangsu, China; 4 Department of Civil Engineering, The University of Hong Kong, Hong Kong, China; Southwest Jiaotong University, CHINA

## Abstract

A common cause of vehicle crashes on urban expressways lies in the unsafe behaviors of drivers. This study focused on analyzing the influence of various unsafe behaviors on crash duration. Based on actual video image of vehicle crashes, 14 unsafe behaviors were identified for the analysis of crashes on urban expressways. Using the correspondence analysis method, the correlation among unsafe behaviors and collision types was obtained. Nonparametric survival analysis was then presented to obtain the survival rate curves of sideswipe crashes and rear-end crashes. Finally, parametric survival analysis method can get the influence of unsafe behaviors on crash duration. The survival rate of any time was quantified through the reasoning of key unsafe behaviors for different types of crashes. The results show that there were striking differences in the duration among different types of crashes. The unsafe behaviors had a significant impact on duration for different types of crashes. This study focused on the duration under the influence of unsafe behaviors before the crash, and the results provide valuable information to prevent crashes, which can improve traffic safety.

## Introduction

Vehicle crashes are a serious problem affecting people’s safety and property. Therefore, how to reduce the crash rate is a hot issue of extreme concern to people around the world. Crashes on urban expressways are more serious than those on other types of urban roads because of the larger volume of traffic and much higher speed on the former [1]. In order to prevent crashes more effectively, it is important to analyze what leads to crashes in the first place, as well as to explore the characteristics of crashes.

Urban expressway crashes are caused by many factors, including the driver [2,3], vehicle [3,4], road [5,6], traffic conditions [3,5,6], and environment [2,3,6,7]. The driver’s behaviour choice while driving is the dominant factor related to the occurrence and the severity of crash [8,9]. When a driver is traveling on an urban expressway, they can choose to change lanes, brake, or use the emergency parking brake in different traffic circumstances from time to time. In this process, if unsafe behavior is displayed, the risk of crashes will increase [10]. The driver’s unsafe behaviors related to crashes on the urban expressway closely, which primarily contribute to traffic crashes [11–13]. Therefore, it is important to study various drivers’ behaviors before the collision.

If a crash occurs on the urban expressway, unsafe behavior is often to blame [8,9] (Shaon et al., 2018a, 2018b). Of course, even if there is unsafe behavior, a crash will not necessarily happen, as the unsafe behaviors can be corrected in time, part of the crashes can be avoided. The timing of unsafe behavior is particularly important.

There are many ways to study unsafe driving behaviors. Some scholars have done research on unsafe behaviors using the traditional method of a questionnaire or a simulated driving experiment [14]. Sullman et al. [15], Han and Zhao [16], and Shirmohammadi et al. [17] studied the crash risk level by a driving simulator. Figueira et al. [18] studied traffic crash risk level by driving simulator. Li et al. [19] used questionnaires and driving simulators to estimate drivers’ lane-change intent. However, there are some limitations to these two methods. There may be an obvious difference in the authenticity between the answer obtained from the actual driving data and from the questionnaire. Moreover, the driving simulation experiment is carried out in virtual conditions, which are significantly different from the real road traffic environment, which is complex and variable.

It is advantageous to study drivers’ unsafe behaviors through crash videos, which help overcome the shortcomings of questionnaires and simulation experiments. The full story of the crash can be successfully reproduced in the video. The crash video gives an insight into the dynamic process of the crash in an actual road environment, and it can be repeatedly reviewed to study the cause of the crash. Some scholars have done some studies about driver’s unsafe behaviors by video on the cause of the crashes, as shown in **Table 1**. However, various types of crash analysis on the urban expressway through video have not been carried out through crash video widely yet, as the video of a crash is difficult to obtain for the appointed road type.

**Table 1:** Research in studying driver’s unsafe behaviors by video.

Author	Time	Video source	Research contents
Piccinini et al. [20]	2017	On-board event data recorders	The factors contributing to commercial vehicle rear-end conflicts
Asadianfam et al. [21]	2020	Monitoring system of big data platform	The drivers’ dangerous behaviors
Seppelt et al. [22]	2017	Video from the Virginia Tech Transportation Institute	The hazardous behaviors of near-crashs
Habibovic et al. [23]	2013	Video data from NDS in Japan	The driving behaviors in human-car crashes
Gu et al. [24]	2019	Unmanned aerial vehicle video data	The drivers’ crash risk at interchange merging areas

By reviewing the video image of crashes, we can determine the sequence of events leading to the crash, the duration of the crash, and the outcome of a variety of unsafe behaviors. It is appropriate to use survival analysis to carry out a multi-factor time-series study. Survival analysis is a statistical analysis method that combines the outcome, influencing factors of events, and duration. At present, survival analysis is widely used in the fields of industry [25], national defense [26], medicine [27], and transportation [28].

Survival analysis has some foundations in the field of traffic safety [29–31]. This method can study the law of survival status and the change trend of survival rate, analyze various influencing factors, and predict the survival outcome. For example, Pawar and Velaga [32] studied the influence of various time pressures on reaction time with this method. Li et al. [33] combined the competitive risk model with the survival analysis model to predict the crash duration. Niu and Ukkusuri [34] used survival analysis to assess different risks of truck drivers engaged in hazardous goods transportation. Liu et al. [35] studied the duration of the punishment period for those who violated road traffic safety rules. Bagloee and Asad [36] studied crash influencing factors at the intersection of CBD using this method. Previous studies have generally focused on the multiple factors that cause a crash. However, it is still not clear how human factors affect the collision type and duration. To fill this research gap, this study aimed to investigate the impact of crash patterns and drivers’ unsafe behaviors on urban expressways using survival analysis.

This paper was organized as follows: in section 2, we extracted the data of crashes and drivers’ unsafe behaviors from the videos and presented a clear definition of each unsafe behavior. In section 3, we introduced the research methods adopted, including survival analysis and correspondence analysis. In section 4, we analyzed the corresponding relationship between crash type and unsafe behaviors. By survival analysis, the influence of unsafe behaviors on crash duration was studied, and discussed the results of the model in detail. The research conclusion was presented in section 5.

### Date

Crash videos were obtained from the public security traffic police department of Hefei in China during the period from 2016 to 2020. In this paper, a total of 223 pieces of video data were collected to understand the complete crash process on the urban expressway. The selected crashes occurred in the main road and ramp area of several urban expressways in Hefei.

### Ethical statement

The study was approved by the public security traffic police department of Hefei and the school of urban construction and transportation at Hefei university. Before implementing the study, our research plan was discussed by several experts. They believed that the research contents would not reveal personal information, nor would it have any negative social impact. As a consequence, they agreed that the research plan was scientifically sound and feasible, and comply with laws and regulations in China.

### Traffic crash classifying

Urban expressway crashes can be classified into the following four types: sideswipe collision, rear-end collision, hit object collision, and other kinds of crashes [37,38]. Other kinds of crashes mainly refer to occasional illegal crashes on the expressway, e.g., illegally reversing vehicles on the expressways or bicyclists driving on the expressways. Sideswipe collisions and rear-end collisions accounted for 83.3% of the total number of urban expressway crashes. These two types of crashes are the most common crashes in urban expressways [39]. These four types of crashes are real crashes that come with the possibility of damaged vehicles, loss of property, or casualties. In contrast, this paper collected and sorted out a total of 103 near-crashes. A near-crash (Seacrist et al. 2020) is not a real crash; there are still unsafe behaviors involved in a near-crash, but the timely correction of the unsafe behaviors prevents the crash from happening. The distribution of each type of crash on urban expressway is shown in **Table 2** below.

**Table 2:** Distribution of various types crashes on urban expressway.

Collision types	Frequency	Ratio	Non-collision	Frequency	Ratio
**Sideswipe**	70	58.3%	Near-crash	103	100%
**Rear-end**	30	25.0%			
**Hit object**	10	8.3%			
**Other types of crashes**	10	8.3%			
**Sum**	120	100%	Sum	103	100%

### Definition of unsafe behaviors

According to the dynamic process of the crash shown in the video, 14 unsafe behaviors were identified in this study. Descriptions of unsafe behaviors are shown in **Table 3**. The 14 unsafe behaviors are identified as follows.

C1, speeding [40]. The design speed of the urban expressway is 80 km/h [41] A vehicle is identified as speeding if its operating speed exceeds 80 km/h.
C2, improper parking. Improper parking is identified as parking unreasonably in places where stopping/parking is prohibited.
C3, straddling lanes without changing lanes. Based on the Road Traffic Safety Law of the People’s Republic of China, it is illegal to drive on traffic markings or shoulders of the road for a long time. In a non-urgent situation, the limit of the time to collision (TTC), which is widely used to evaluate safety for sideswipe collisions, is 5.5 s [42]. When the span exceeds 5.5 s, in a non-urgent situation, the vehicle is going straight without lane-changing behavior, and the turn signal is not turned on. This behavior is identified as straddling lanes.
C4, conflict driving behavior. Driving in a dangerous or an aggressive way. The concrete manifests of this behavior include that a vehicle driver tailgates another vehicle and forces it to stop and get out of the way, speed up to prevent another vehicle from overtaking, flash their high beams inappropriately, honk inappropriately, or make rude gestures to other drivers [43].
C5, continuous changing more than one lane at a time. It is illegal to change lanes more than one lane at a time.
C6, driving into a forbidden area. Vehicles are not allowed to drive into some traffic channelized zones or emergency vehicle lanes in the scope of urban expressways.
C7, close following. Under uncongested road conditions, this condition applies when a vehicle follows another vehicle with a distance less than a vehicle length, and it lasts for more than 5.5 [44].
C8, unsafe passing. In the off-ramp driving process, a vehicle accelerates off the main line and into the ramp suddenly, slams on the brakes, or backs up.
C9, unsafe merging. In the on-ramp driving process, a driver fails to give priority to the main road vehicles.
C10, failure to turn on hazard warning lights. A driver fails to turn on the hazard warning lights in emergency situations, for example, when the visibility is less than 100 meters, the vehicle malfunctions, a crash occurs, or the vehicle temporarily parks on the roadside.
C11, failure to turn on signal when changing lanes. A driver fails to turn on the turn signal in advance when he makes a turn, changes a lane, prepares to overtake, or leaves a parking place.
C12, queue-jumping. To define the queue-jumping behavior, the following rules should be met. The initial horizontal distance between a lane-changing vehicle and a straight-moving vehicle is less than 2.2 m. The maximum lateral acceleration of the straight-moving vehicle is less than 0.07 g, and lane offset is less than 1.0 m. The maximum length of lane-change process is no more than 75 m. The velocity of both lane-changing and straight-moving vehicles should be more than 1 m/s [42].
C13, distracted and inattentive driving [1]. Distracted and inattentive driving refers to behavior leading to missed observations of some kind, which in turn leads to a critical event of ‘timing’ (premature, late action, or no action) or (incorrect) ‘direction’. When the driver lacks motivation to carry out their task in the best way possible, an object or sequence of events diverts the driver’s attention, or the driver is used to ascertaining the environment makes it difficult to discover changes [45]. The specific performance of this behavior includes the driver’s failure to reduce speed in time or improper driving behavior in an emergency. The discriminant method of driver’s failure to reduce speed in time is that, accounting for possible unfavorable driving situation, the vehicle maximum deceleration is set for 10 m/s2 and 8 m/s2 for dry and wet roads, respectively [46]. When the vehicle starts to decelerate until it stops, the maximum deceleration does not reach the value [46]. The vehicle is considered to fail to reduce speed in time. Improper driving behavior in an emergency is another form of distracted and inattentive driving. This unsafe behavior means that a driver improperly operates the vehicle when a vehicle in front changes a lane, which leads to more vehicles being involved in a crash.
C14, lane change without checking the rearview mirror or not scanning the road around. Before changing a lane, the driver should observe the situation from the rearview mirror in advance to understand the situation on both sides of the car and behind. Making lane changes without checking the rearview mirror or not scanning the road around is an unsafe behavior.

**Table 3:** The table of unsafe behaviors on urban expressway.

Number	Unsafe behavior	Frequency	Percentage
**C1**	Speeding	4	0.8%
**C2**	Improper parking	15	3.1%
**C3**	Straddling lanes without changing lanes	21	4.4%
**C4**	Conflict driving behavior	25	5.2%
**C5**	Continuous changing more than one lane at a time	32	6.7%
**C6**	Driving into a forbidden area	41	8.5%
**C7**	Close following	10	2.1%
**C8**	Unsafe passing	35	7.3%
**C9**	Unsafe merging	20	4.2%
**C10**	Failure to turn on hazard warning lights	10	2.1%
**C11**	Failure to turn on signal when changing lanes	62	12.9%
**C12**	Queue-jumping	82	17.0%
**C13**	Distracted and inattentive driving	72	15.0%
**C14**	Lane change without checking the rearview mirror or not scanning the road around	57	11.9%

In all crashes, 486 unsafe behaviors are recognized, as shown in Table 3. There is at least one unsafe behavior behind every urban expressway crash, including near crashes. The average number of unsafe behaviors in an urban expressway crash is 2.18. It can be concluded that unsafe behaviors are closely related to crashes on urban expressway.

### Definition of crash duration

The crash duration is the period from the beginning of the crash trend to the moment when the crash happens. For example, for a sideswipe collision, the crash duration is the period from the moment when a vehicle shows the lane change trend to the moment when a crash occurs. For a rear-end collision, the crash duration refers to the period from the moment when the vehicle in front stops to the moment when the vehicle behind fails to slow down and brake in time, and then there is a collision between vehicles. For a hit object collision, the crash duration is the period from the moment when a vehicle shows the lane change trend to the moment when a crash occurs between a vehicle and a fixed object. For a near-crash, the duration has two cases. One of the cases is that the duration is between the time for a front vehicle beginning slowing down and the time when a rear-end crash is likely to occur. Alternatively, the other case is that the duration starts when a vehicle has a tendency to change lanes, and it ends when a sideswipe collision is likely to occur. The crash duration of each type could be displayed according to the video.

## Research methodology

### Survival analysis

Survival analysis is a statistical analysis method that studies the relationship among duration, outcome and numerous influencing factors. The outcome in this paper corresponds to an expressway crash happening. The corresponding crash duration is the period from the normal driving state to the occurrence of the crash. And various unsafe behaviors are taken as the influencing factors. It is more consistent with the research content of this paper to use the method of survival analysis.

There are three types of survival analysis: nonparametric analysis, semi-parametric analysis, and parametric analysis [47,48]. Nonparametric analysis can estimate the survival function and compare two or more groups of survival distribution functions. While this method ignores the influence of variables. Besides, this model cannot give the risk rate at each time point [49]. Semi-parametric analysis is represented the relationship between influencing variables and survival rate. The limitation of this method is the distribution of time (and risk function) is not defined [46]. Parametric models can be used for multivariate survival analysis, and they can better match the actual crashes [47]. When the distribution of survival time is known, the parametric analysis method is more accurate and effective than the non-parametric analysis method and semi-parametric analysis method [32]. This paper uses two methods, nonparametric analysis and parametric analysis, to analyze the mechanism of crashes.

The Kaplan–Meier method (KM) [50,51] is the most commonly used nonparametric estimation method. Assume that there are n samples and every sample has a duration of *i* (*i≤n*). In this study, the sample refers to each urban freeway crash, including near-crash. Sort samples by pre-set time period *m*_*x*_, and *m*_*x*_ refers to the longest duration of crash videos observed. The estimate of the survival rate *S*(*t*) can be expressed as follows:

$$S(t)=\prod_{t_{i}\leq m_{x}}\frac{n_{i}–d_{i}}{n_{i}}$$ (1)

Where, *m*_*x*_ is a period of time; *t*_*i*_ is each simple duration belonging to *m*_*x*_; *n*_*i*_ is the number of all samples in this period; *d*_*i*_ is the number of failed samples in this period; *S*(*t*) is the survival rate in this period.

The accelerated failure model (AFT) [29] is a type of parameter estimation of survival analysis. It can judge the influence of unsafe behaviors on different types of crashes. When the distribution of duration is known, we can study how the explanatory variables affect survival rate.


$$\log(t)=g_{1}*z_{1}+g_{2}*z_{2}+\ldots +\omega_{i}$$ (2)

Where, *t* is the survival time of sample; *z*_*i*_ is unsafe behavior covariant; *g*_*i*_ is the coefficient of covariant for *z*_*i*_;*ω*_*i*_ is the random error.

The distribution of survival data was fitted. In this paper, curve fitting was conducted for the duration of the sideswipe collision and rear-end collision. According to statistics of crash duration from video image content, the Weibull curve distribution was found to be a good fit. Therefore, the Weibull distribution was selected as survival distribution form in this study. The risk function and survival function under a certain condition is shown as follows [52].


$$\gamma =\frac{1}{p}$$ (3)

$$h(t|x)=(\lambda P){\left(\lambda t\right)}^{P-1}$$ (4)

$$S(t)=\exp(-\lambda t^{\gamma })$$ (5)

Where, h(t|x) is the risk function under a certain condition; S(t|x) is the survival function under a certain condition; *λ* is the scale parameter; *P* is the shape parameter that determines the mutative risk with time. *λ* can be condensed into the following form:

$$\lambda =\exp[-(g_{1}\times z_{1}+g_{2}\times z_{2}+\ldots +w_{i})*\gamma ]$$ (6)

### Correspondence analysis

Correspondence analysis is a low-dimensional graphical representation used to find associations between rows and columns of a contingency table [53]. It can also reveal the differences among all types of events. By projecting all parameters of each row and column into a two-dimensional Euclidean space, the relationship of each row and column can be kept to the maximum extent. The process of correspondence analysis is as follows.

1. Each unsafe behavior of every crash is obtained and represented by bivariate variables, listed in the matrix, and obtain the corresponding contingency table.
2. Divide *n* by all the elements to get the corresponding matrix.
$$p=p_{ij}=n_{ij}/n$$ (7)
$$r=(p_{1\cdot },p_{2\cdot },\cdots ,p_{p\cdot })\prime$$ (8)
$$c\prime =(p_{\cdot 1},p_{\cdot 2},\cdots ,p_{\cdot q})$$ (9)
  Where, **p** is the frequency matrix; **r** represents the last column in the table; **c’** represents the last row in the table.
3. The frequency diagonal matrix is shown below:
$$D_{r}=\mathrm{diag}(p_{1\cdot },p_{2\cdot },\cdots ,p_{p\cdot })$$ (10)
$$D_{c}=\mathrm{diag}(p_{\cdot 1},p_{\cdot 2},\cdots ,p_{\cdot q})$$ (11)
  Where, D_r_ is row contour matrix; D_c_ is column contour matrix.
4. Get the total inertia. The total inertia can be used as a measure of the correlation between row and column variables, such as the expression below.
$$The\;total\;inertia=\frac{\chi^{2}}{n}=\sum_{i=1}^{p}\sum_{j=1}^{q}\frac{{\left(p_{ij}-p_{i\cdot }p_{\cdot j}\right)}^{2}}{p_{i\cdot }p_{\cdot j}}$$ (12)
5. The diagram of correspondence analysis. A plane coordinate is constituted by the row contours and a plane coordinate is constituted by the column contours, forming a two-dimensional correspondence analysis diagram. The cumulative contribution rate of total inertia is judged by correspondence analysis diagram. If the value is large, it means that the corresponding analysis diagram could explain nearly all the variations of the total variables in the table. Correspondence analysis method can also visually show the correlation between various variables.

## Analysis and results

### Corresponding analysis

**Fig 1:**
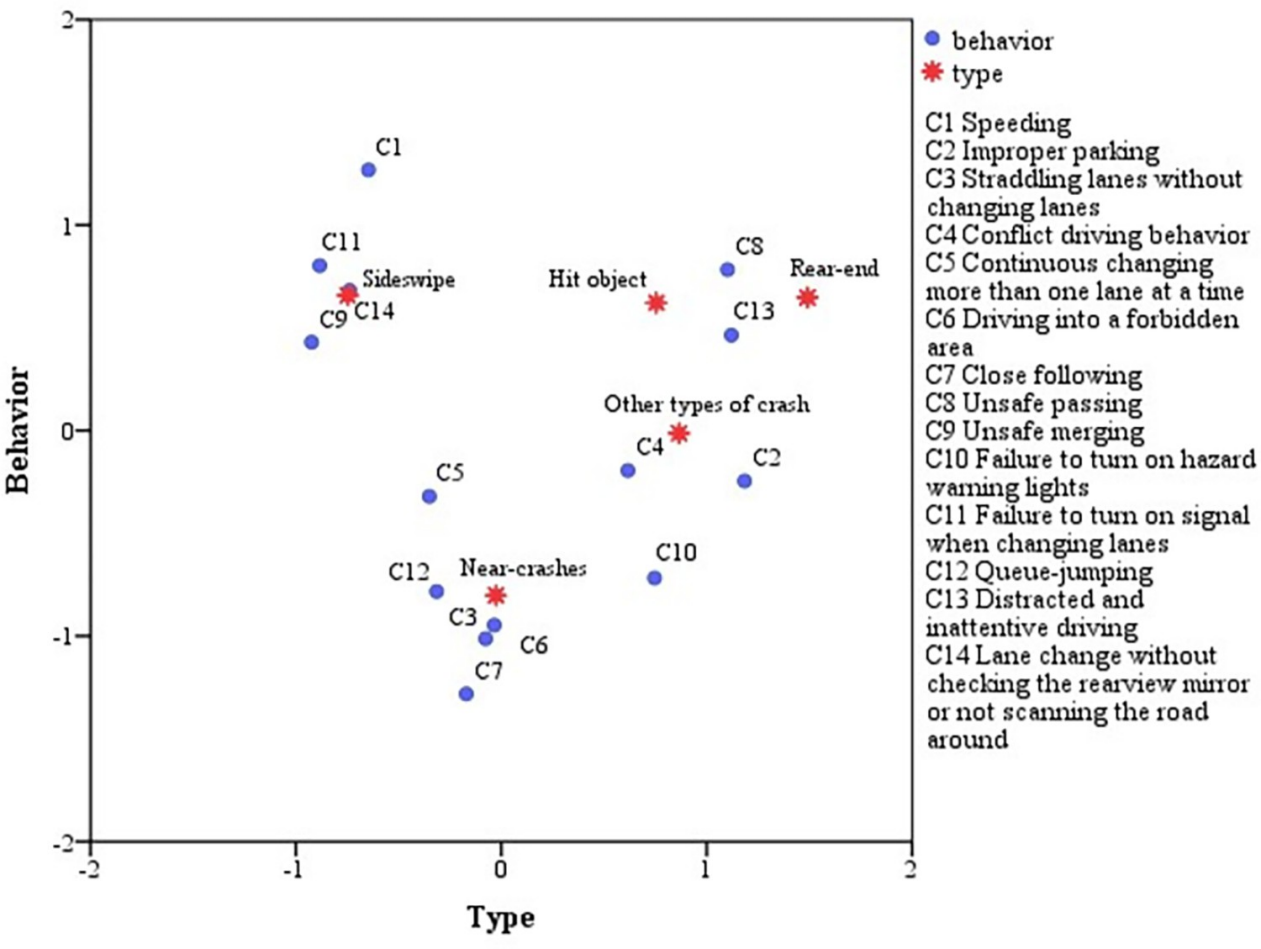
Corresponding analysis of unsafe behaviors for different types of crashes.

For different types of urban expressway crashes, the unsafe behaviors causing crashes are also different. According to correspondence analysis, the relationship between different types of crashes and unsafe behaviors can be clearly seen, as shown in **Fig 1** below. The final corresponding relation between crash types and unsafe behaviors is shown in **Table 4**.

**Table 4:** The corresponding relationship table between crash types and unsafe behaviors.

Types	C9	C11	C14	C1	C5	C12	C7	C3	C6	C4	C10	C8	C13	C2	Average
**Sideswipe**	**14**	**48**	**40**	3	**12**	**21**	1	3	5	4	0	9	11	0	12
**Near-crash**	6	10	12	0	**18**	**59**	9	**16**	**32**	12	7	6	**18**	8	15
**Hit object**	0	1	**2**	1	0	0	0	0	**3**	0	0	1	**8**	0	1
**Other types of crashes**	0	1	0	0	0	1	0	**2**	0	**2**	0	0	**5**	0	1
**Rear-end**	0	2	3	0	2	1	0	0	1	**7**	3	**19**	**30**	**7**	**5**

As can be seen from the results, shown in **Table 4**, the corresponding relationship between crash types and unsafe behaviors is obtained. The bigger the number, the stronger the correlation. In each group of types of crashes, unsafe behavior variables greater than the average value are selected. The sideswipe collision is associated with five variables: C5 (continuous changing more than one lane at a time), C9 (unsafe merging), C11 (failure to turn on signal when changing lanes), C12 (queue- jumping), C14 (lane change without checking the rearview mirror or not scanning the road around). Of these, C9 (unsafe merging), C11 (failure to turn on signal when changing lanes), C12 (queue-jumping), C14 (lane change without checking the rearview mirror or not scanning the road around) are displayed by the driver while steering. Most crashes occur largely because the drivers do not use their turn signals correctly to give an explicit prompt to vehicles in adjacent lanes. In other cases, crashes are mostly caused by the unsafe behavior of queue-jumping. That is because that the driver does not look for the appropriate gap to change lanes, or does not observe the situation of the adjacent lane. Wang et al. [42] analyzed queue-jumping behavior on urban expressway and drew the same conclusion. These errors are an important cause of crashes.

The unsafe behaviors that cause rear-end collision are different from those of the sideswipe collisions. C2 (improper parking), C4 (conflict driving behavior), C8 (unsafe passing), C13 (distracted and inattentive driving) are the main unsafe behaviors that induce rear-end collisions on urban expressways. Piccinini et al. [20] came to the same conclusion, while in this paper, the classification of unsafe behaviors is more detailed. The rear-end collision is mainly caused by the unsafe behaviors of followers, as the driver does not adjust the state fast enough to respond to emergencies ahead.

The unsafe behaviors that cause hitting object crashes are as follows, and they are C6 (driving into a forbidden area), C13 (distracted and inattentive driving), C14 (lane change without checking the rearview mirror or not scanning the road around). This kind of collision is mainly caused by distracted driving, leading to the vehicle hitting the dividing medians or guards on either side of the urban expressway.

For other types of crashes on urban expressways, C3 (straddling lanes without changing lanes), C4 (conflict driving behavior), C13 (distracted and inattentive driving) are the main behaviors that induce crashes. The number of such crashes is small.

Near-crash events are associated with six unsafe behaviors: C3 (straddling lanes without changing lanes), C5 (continuous changing more than one lane at a time), C6 (driving into a forbidden area), C13 (distracted and inattentive driving).

### Nonparametric survival analysis

**Fig 2:**
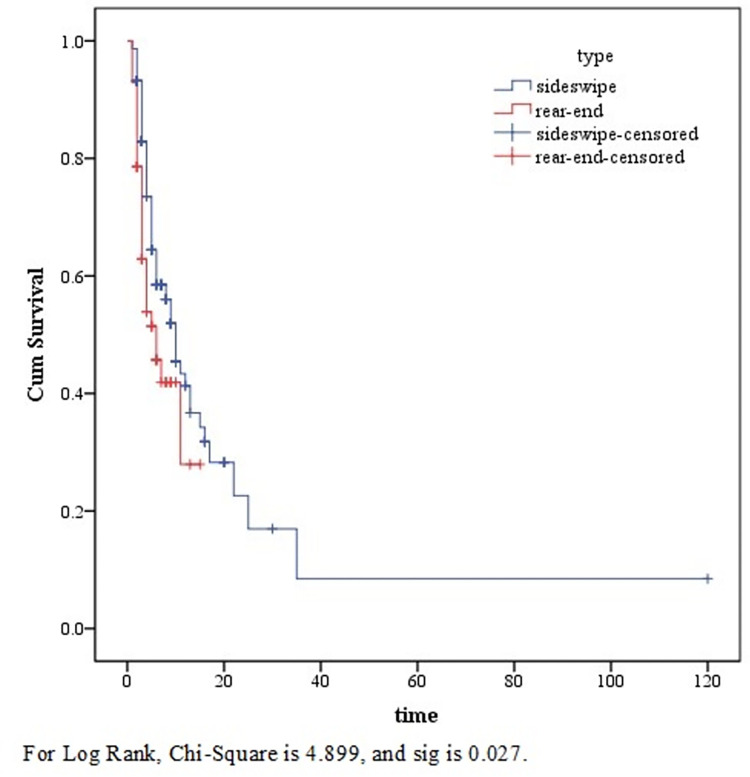
The figure of non-parametric survival analysis method.

This study took the sideswipe collision and rear-end collision to do survival analysis research. These two types of crashes are the most common crashes in urban expressways [1]. The other two types of crashes accounted for a small proportion and did not do survival analysis. Besides, near-crashes were introduced for comparative analysis. For non-parametric survival analysis, this study used the KM method. This method can estimate the survival probability of the crash based only on the duration and the type of crash, instead of other variables. 203 samples and their crash duration were obtained for analysis, including sideswipe collisions, rear-end collisions, and near-crashes. The results are shown in **Fig 2**. For the log-rank test, the chi-square is 4.899, and the significance (sig) is 0.027. This means that the duration of these two types of crashes is significantly different, so we should deal with them separately.

However, the KM curve provides discrete estimation of survival probability rather than successive value. In addition, this method lacks the analysis of the crash process, so it is impossible to know the influence of unsafe behaviors on the crash. In view of the above problems, the parametric survival model could be used for further analysis in this study. The unsafe behaviors corresponding to different crash types were taken as research parameters.

### Parametric survival analysis

The Weibull AFT model was used to study the influence of each unsafe behavior parameter on the risk of two types of crashes. The results of the sideswipe collision are shown in **Table 5**, and the results of the rear-end collision are shown in **Table 6**. The estimate for each parameter, standard error (SE), 95% confidence limit, P-value, and shape parameter (P) are obtained from the analysis results. As the two tables shown, for both types of crashes, the shape parameter P is greater than 1, which shows that the risk rate increases with time. With the increase in crash duration, the survival probability of the crash decreases, which is consistent with the actual road crash situation.

**Table 5:** Analysis results of sideswipe collision.

Variable	Estimate	SE	95% confidence limit		P-value
**C9**	1.329	0.559	0.232	2.425	0.018**
**C11**	-0.753	0.461	-1.657	0.151	0.103
**C14**	2.449	0.477	1.513	3.386	0.000***
**C12**	4.190	0.474	3.260	5.121	0.000***
**C5**	2.395	0.599	1.220	3.569	0.000***
**log(scale)**	0.714	0.062	0.591	0.837	0.000***
**P (** **shape** **)**	2.042				

* for P value ≤ 0.1.

**for P value ≤ 0.05.

*** for P value ≤ 0.01.

**Table 6:** Analysis results of rear-end collision.

Variable	Estimates	SE	95% confidence limits		P-values
**C4**	2.721	0.895	0.966	4.475	0.002***
**C8**	0.814	0.589	-0.340	1.970	0.167
**C2**	2.444	1.253	-0.012	4.901	0.051*
**C13**	1.813	0.430	0.969	2.657	0.000***
**log(scale)**	1.099	0.067	0.967	1.232	0.000***
**P (** **shape** **)**	3.004				

* for P value ≤ 0.1.

**for P value ≤ 0.05.

*** for P value ≤ 0.01.

In the sideswipe collision, C5 (continuous changing more than one lane at a time), C9 (unsafe merging), C12 (queue-jumping), C14 (lane change without checking the rearview mirror or not scanning the road around) are the four main factors that have a significant influence on the crash duration. In the AFT model, the factors that have a significant influence on duration have a reduction effect, so the risk of crash will increase accordingly. The influence degree of three unsafe behaviors on duration is also different. In particular, C12 (queue-jumping) increase the risk by 319%, compared with not having this behavior, which make them the most obvious influence. Accordingly, when C14 (lane change without checking the rearview mirror or not scanning the road around) or C5 (continuous changing more than one lane at a time) occurs, the risk rises by 144.9% or 139.5% higher than having displaying no such behavior. C9 (unsafe merging) increases by 32.9% compared with those crashes without such behavior.

In a rear-end crash, there are many key factors affecting the duration, such as C2 (improper parking), C4 (conflict driving behavior) and C13 (distracted and inattentive driving). These behaviors are positively correlated to the risks of crashes. C4 (conflict driving behavior) increases by 172.1% compared with those crashes without such behavior, so it has the most obvious impact of all the behaviors. Correspondingly, when C2 (improper parking) or C13 (distracted and inattentive driving) occurs, 144.4% and 81.3% increases in crash risk, respectively.

**Fig 3:**
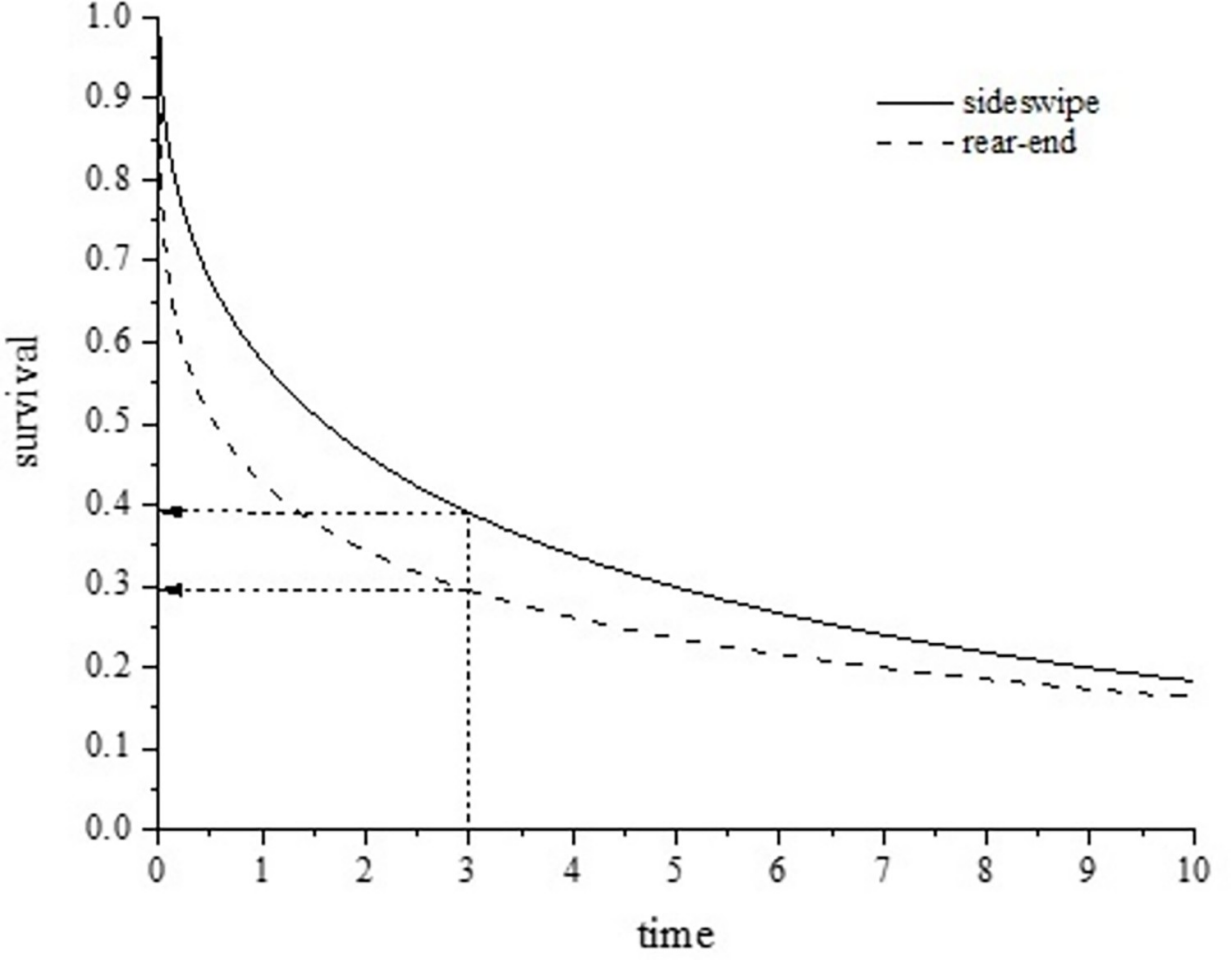
The figure of parametric survival analysis method.

In the Weibull-AFT model, when considering the influence of various unsafe behaviors on different types of crashes, it is easy to obtain the survival rate curves of two types of crashes at any moment. The results are shown in **Fig** 3. As can be seen from the figure, when unsafe behaviors are taken into account, the distinction of these two types of accidents is more obvious, and there is no overlap between them at any time. For the same survival rate, the duration of rear-end collision was shorter than that of sideswipe collision, and the cumulative survival rate of sideswipe collision was higher than that of rear-end collision.

We went even further to get the estimated value of the survival rate at any time. Based on survival function, the crash survival rate for both types of crashes could be obtained. For example, when t = 3 s, the survival rate of the sideswipe collision is 39.06%. The survival probability of rear-end collision is 29.52%. At the same duration, the survival rate of a rear-end collision is lower than that of the sideswipe collision.

**Fig 3** shows how the survival curves of the two types of crashes change with time. For sideswipe collisions, the survival rate declines more rapidly within 6 s. The cumulative survival curve has an inflection point at 6 s, the survival rate decreased rapidly between 0 and 6 s. It is indicated that for most sideswipe collisions, the duration is within 6 s. For rear-end crashes, the cumulative survival curve has an inflection point at 4 s. During the 4 s, the survival rate decreases rapidly with time. It is indicated that for most rear-end collisions, the duration is within 4 s. For a driver, the correction of unsafe behavior needs to be completed within a short time. Rear-end collisions are more difficult to avoid than sideswipe collisions.

In contrast to analyses of sideswipe collision and rear-end collision, when a sideswipe collision occurred, this was mainly due to having no time to correct unsafe behaviors (e.g., continuous changing more than one lane at a time, unsafe merging, queue-jumping, lane change without checking the rearview mirror or not scanning the road around). If the duration was longer than 6 s, there was some time for a driver to correct unsafe behaviors, the risk was less, and the accident rate was lower accordingly. For a rear-end collision, unsafe behaviors (e.g., improper parking, conflict driving behavior, distracted and inattentive driving) occurred in 4 s timeframe were to be given adequate attention, which had a great influence on the accident rate.

## Conclusion

Unsafe behavior analysis is a hot topic in the field of traffic safety and keeps developing. In this study, 14 common unsafe behaviors related to urban expressway crashes were identified. To explore traffic unsafe behaviors that lead to crashes, correspondence analysis was used to find correlations between unsafe behaviors and crash types. Subsequently, we used survival analysis to study unsafe behaviors on each crash type, qualitatively and quantitatively. This part focused on the two most common types of crashes on urban expressways: sideswipe collisions and rear-end collisions.

Sorting out unsafe behaviors on urban expressways. The classification and definition of unsafe behavior in this paper are different from previous literature. The unsafe behaviors studied in this paper are studied from the driver’s first perspective, which can more accurately and intuitively display and restore the driver’s behavioral factors of the collision, and this factor often makes the greatest contribution to the collision.

Important conclusions are summarized in the following. For different types of crashes on urban expressways, the unsafe behaviors that induced crashes were also different. According to correspondence analysis, different unsafe behaviors will affect different types of urban expressway collisions. One type of collision is mainly affected by 3–5 main unsafe behaviors. The same unsafe behavior can affect different types of collisions, such as C13. For different types of collisions avoidance, the types of unsafe behaviors can be more clearly defined. There are clearer guidelines for accident risk avoidance.

The analyses conducted with the survival analysis methodology yielded a clear picture of the main behavior patterns causing crashes on urban expressways. The duration varied in different types of collisions. Unsafe behaviors could affect the duration. For the same survival rate, the crash duration of a rear-end collision was less than that of a sideswipe collision. We could also accurately identify the crash survival rate at any time t based on unsafe behaviors of great significant impact.

For a sideswipe collision, the survival rate decreases rapidly in 6 s. The drivers could correct unsafe behaviors during this time. For a rear-end collision, unsafe behaviors that occurred within a 4 s timeframe were to be given adequate attention, which had a great influence on the crash rate.

The video image displayed before the crash was more conducive to preventing urban expressway crashes from the source, which was more active and effective than the prevention after the crash. The analysis results of this paper are instructive for us to understand the characteristics of expressway crashes, which can be used in various fields, such as traffic crash safety prevention, traffic management, traffic policy making, and other fields. Future research should further expand the sample size of crashes. As this study focused solely on sideswipe collisions and rear-end collisions, further research is recommended on the impact of various types of crashes on urban expressways.

## References

[1] Zhang H, Li SY, Wu CZ, Zhang Q, Wang YF, (2020). Predicting crash frequency for urban expressway considering collision types using real-time traffic data. Journal of Advanced Tranportaion, id 8523818. 10.1155/2020/8523818.

[2] Yamamotoa T, Hashiji J, Shankar VN, (2008). Underreporting in traffic accident data, bias in parameters and the structure of injury severity models. Accident Analysis and Prevention, 40(4), 1320–1329. doi: 10.1016/j.aap.2007.10.016 18606262

[3] Mannering F, Shankar V, Bhat CR (2016). Unobserved heterogeneity and the statistical analysis of highway accident data. Analytic Methods in Crash Research, 11, 1–16. 10.1016/j.amar.2016.04.001.

[4] Hosseinzadeh A, Moeinaddini A, Ghasemzadeh A (2021). Investigating factors affecting severity of large truck-involved crashes: Comparison of the SVM and random parameter logit model. 10.1016/j.jsr.2021.02.012. 34092305

[5] Rj Yu, Wang YY, Quddus M, Li J, Wang XS, Tian Y, (2021). Investigating vehicle roadway usage patterns on the Shanghai urban expressway system and their impacts on traffic safety International. Journal of Sustainable Transportation, 15(3), 217–228. 10.1080/15568318.2020.1722869.

[6] Pei X, Sze NN, Wong C, Yao DY, (2016). Bootstrap resampling approach to disaggregate analysis of road crashes in Hong Kong. Accident Analysis and Prevention, 95, 512–520. doi: 10.1016/j.aap.2015.06.007 26164706

[7] Uttl B, Kisinger k (2010). Meaning of missing values in eyewitness recall and accident records. Plos One, 5 9(5), e12539. doi: 10.1371/journal.pone.0012539 20824054 PMC2932730

[8] Shaon MRR, Qin X, Chen Z, Zhang J, (2018a). Exploration of contributing factors related to driver errors on highway segments. Transportation Research Record, 2627(38), 22–34. 10.1177/0361198118790617.

[9] Shaon MRR, Qin X, Shirazi M, Lord D, Geedipally SR, (2018b). Developing a random parameters negative binomial-lindley model to analyze highly over-dispersed crash count data. Analytic Methods in Accident Research, 18, 33–44. 10.1016/j.amar.2018.04.002.

[10] Yuan YL, Yang M, Guo YY, Rasouli S, Gan ZX, Ren YF, (2021). Risk factors associated with truck-involved fatal crash severity: Analyzing their impact for different groups of truck drivers. Journal of Safety Research, 76, 154–165. doi: 10.1016/j.jsr.2020.12.012 33653546

[11] Wu YW, Hsu TP (2021). Mid-term prediction of at-fault crash driver frequency using fusion deep learning with city-level traffic violation data. Accident Analysis and Prevention, 150, 105910. doi: 10.1016/j.aap.2020.105910 33302233

[12] Zhang W, Cao J, Xu J, (2017). How to quantitatively evaluate safety of driver behavior upon accident? A biomechanical methodology. Plos One, 12(12), e0189455. doi: 10.1371/journal.pone.0189455 29240789 PMC5730198

[13] Shen BY, Qu WN, Ge Y, Sun XH, Zhang K, (2017). The relationship between personalities and self-report positive driving behavior in a Chinese sample. Plos One, 13(1), e0190746. 10.1371/journal.pone.0190746. PMC5764283 29324823

[14] Üzümcüoglu Y, Özkan T, Wu CZ, Zhang H, (2020). Traffic climate and driver behaviors: The moderating role of driving skills in Turkey and China. Journal of Safety Research, 75,87–98. doi: 10.1016/j.jsr.2020.08.004 33334497

[15] Sullman MJM, Stephens AN, Taylor JE, (2019). Dimensions of aberrant driving behaviour and their relation to crash involvement for drivers in New Zealand. Transportation Research Part F, 66 111–121. 10.1016/j.trf.2019.08.024.

[16] Han WL, Zhao JY, (2020). Driver behaviour and traffic accident involvement among professional urban bus drivers in China. Transportation Research Part F, 74, 184–197. 10.1016/j.trf.2020.08.007.

[17] Shirmohammadi H, Hadadi F, Saeedian M, (2019). Clustering analysis of drivers based on behavioral characteristics regarding road safety international. Journal of Civil Engineering, 17(8A), 1327–1340. 10.1007/s40999-018-00390-2.

[18] Figueira AC, Larocca APC, (2013). Proposal of a driver profile classification in relation to risk level in overtaking maneuvers. Transportation Research Part F-Traffic Psychology and Behavior, 74,375–385. 10.1016/j.trf.2020.08.012.

[19] Li XH, Wang WS, Roetting M, (2019). Estimating driver’s lane-change intent considering driving style and contextual traffic. IEEE Transactions on Intelligent Transportation Systems, 20(9), 3258–3271. 10.1109/TITS.2018.2873595.

[20] Piccinini GB, Engstrom J, Bargman J, Wang XS, (2017). Factors contributing to commercial vehicle rear-end conflicts in China: a study using on-board event data recorders. Journal of Safety Research, 62, 143–153. doi: 10.1016/j.jsr.2017.06.004 28882261

[21] Asadianfam S, Shamsi M, Kenari AR, (2020). Big data platform of traffic violation detection system: identifying the risky behaviors of vehicle drivers. Multimedia Tools and Applications, 79(33–34), 24645–24684. 10.1007/s11042-020-09099-8.

[22] Seppelt BD, Seaman S, Lee J, Angell LS, Mehler B, Reimer B, (2017). Glass half-full: on-road glance metrics differentiate crashes from near-crashes in the 100-Car data. Accident Analysis and Prevention, 107,48–62. doi: 10.1016/j.aap.2017.07.021 28787612

[23] Habibovic A, Tivesten E, Uchida N, Bargman J, Aust ML, (2013). Driver behavior in car-to-pedestrian incidents: An application of the Driving Reliability and Error Analysis Method (DREAM). Accident Analysis and Prevention, 50, 554–565. doi: 10.1016/j.aap.2012.05.034 22749319

[24] Gu X, Abdel-Aty M, Xiang QJ, Cai Q, Yuan JH, (2019.) Utilizing UAV video data for in-depth analysis of drivers’ crash risk at interchange merging areas. Accident Analysis and Prevention, 123, 159–169. doi: 10.1016/j.aap.2018.11.010 30513457

[25] Fritsch M, Brixy U, Falck O, (2006). The effect of industry region and time on new business survival–a multi-dimensional analysis. Review of Industrial Organization, 28(3), 285–306. 10.1007/s11151-006-0018-4.

[26] Alexander M, Zhu KM, Cullen J, Byrne C, Brown D, Shao S, et al, (2019). Race and overall survival in men diagnosed with prostate cancer in the Department of Defense Military Health System, 1990–2010. Cancer Causes & Control, 30(6), 627–635. doi: 10.1007/s10552-019-01163-5 30997591

[27] Tsimberidou AM, Hong DS, Wheler JJ, Falchook GS, Janku F, Naing A, et al, (2019). Long-term overall survival and prognostic score predicting survival: the IMPACT study in precision medicine. Journal of Hematology & Oncology, 12(1), pp 12. doi: 10.1186/s13045-019-0835-1 31888672 PMC6937824

[28] Choudhary P, Velaga NR, (2017a). Modelling driver distraction effects due to mobile phone use on reaction time. Transportation Research Part C, 77, 351–365. 10.1016/j.trc.2017.02.007.

[29] Haque M, Washington S, (2014). A parametric duration model of the reaction times of drivers distracted by mobile phone conversations. Accident Analysis and Prevention, 62, 42–53. 10.1016/j.aap.2013.09.010. 10.1016/j.aap.2013.09.010. 24129320

[30] Wu LT, Meng Y, Kong XQ, Zou YJ, (2020). Incorporating survival analysis into the safety effectiveness evaluation of treatments: Jointly modeling crash counts and time intervals between crashes. Journal of Transportation Safety & Security. 108, 1–21. 10.1080/19439962.2020.1786871.

[31] Zou YJ, Lin B, Yang XX, Wu LT, Abid MM, Tang JJ, (2021). Application of the bayesian model averaging in analyzing freeway traffic incident clearance time for emergency management. Journal of Advanced Transportation. Id: 6671983, 9 pages. 10.1155/2021/6671983.

[32] Pawar NM, Velaga NR, (2020). Modelling the influence of time pressure on reaction time of drivers. Transportation Research Part F, 72, 1–22. 10.1016/j.trf.2020.04.017.

[33] Li RM, Pereira FC, Ben-Akiva ME, (2015). Competing risks mixture model for traffic incident duration prediction. Accident Analysis and Prevention, 54, 192–201. doi: 10.1016/j.aap.2014.11.023 25485730

[34] Niu SF, Ukkusuri SV, (2020). Risk assessment of commercial dangerous -goods truck drivers using geo-location data: a case study in China. Accident Analysis and Prevention, 137, id:105427. doi: 10.1016/j.aap.2019.105427 32032934

[35] Liu ZY, Wu HB, Li RM, (2020). Effects of the penalty mechanism against traffic violations in China: A joint frailty model of recurrent violations and a terminal accident. Accident Analysis and Prevention, 141, id:105547. doi: 10.1016/j.aap.2020.105547 32334154

[36] Bagloee SA, Asad Mi, (2016). Crash analysis at intersections in the CBD: a survival analysis model. Transportation Research Part A, 94, 558–572. 10.1016/j.tra.2016.10.019.

[37] Xu C, Wang XS, Yang H, Xie K, Chen XH, (2019). Exploring the impacts of speed variances on safety performance of urban elevated expressways using GPS data Accident Analysis and Prevention, 123, 29–38. 10.1016/j.aap.2018.11.012. 30458332

[38] Zhang CJ, He J, Yan XT, Liu ZY, Chen YK, Zhang H, (2021). Exploring relationships between microscopic kinetic parameters of tires under normal driving conditions, road characteristics and accident types. Journal of Safety Research, 78, 80–95. doi: 10.1016/j.jsr.2021.05.010 34399934

[39] Zhang Z, Guo YS, Fu R, Yuan W, Wang C, (2020). Linking executive functions to distracted driving, does it differ between young and mature drivers? Plos One, 15(9), e0239596. doi: 10.1371/journal.pone.0239596 32970738 PMC7514019

[40] Ward NJ, Finley K, Otto J, Kack D, Gleason R, Lonsdale T, (2020). Traffic safety culture and prosocial driver behavior for safer vehicle-bicyclist interactions. Journal of Safety Research, 75, 24–31. doi: 10.1016/j.jsr.2020.07.003 33334482

[41] Code for design of urban road engineering, CJJ 37–2019. Ministry of Housing and Urban-Rural Developmeng of the People’s Republic of China.

[42] Wang XS, Yang MM, Hurwitz D, (2019). Analysis of cut-in behavior based on naturalistic driving data. Accident Analysis and Prevention, 124, 127–137. doi: 10.1016/j.aap.2019.01.006 30639685

[43] Houston JM and Harris P (2003). The aggressive driving behavior scale: developing a self-report measure of unsafe driving practices. North American Journal of Psychology, 5(2), 269–278.

[44] Berg TGCVD, Kroesen M, Chorus CG, (2020). Does morality predict aggressive driving? A conceptual analysis and exploratory empirical investigation. Transportation Research Part F, 74, 259–271. 10.1016/j.trf.2020.08.017.

[45] Talbot R, Fagerlind H, Morris A, (2013). Exploring inattention and distraction in the safetynet crash causation database. Accident Analysis and Prevention, 60, 445–455. 10.1016/j.aap.2012.03.031. 24176106

[46] Xiong XX, Wang M, Cai YF, Chen L, Farah H, Hagenzieker M, (2019). A forward collision avoidance algorithm based on driver braking behavior. Accident Analysis and Prevention, 129, 30–43. doi: 10.1016/j.aap.2019.05.004 31103877

[47] Fu HQ, Wilmot CG, (2006). Survival analysis-based dynamic travel demand models for hurricane evacuation. Transportation Research Record-Series, 1964, 211–218.

[48] John O’Quigley, (2021). Department of statistical science, University College London, London WC1E 6BT, UK. Survival Analysis. ISBN 978-3-030-33438-3.

[49] Nan S, Yan L, Tu R, Li T, (2021). Modeling lane-transgressing behavior of e-bike riders on road sections with marked bike lanes: a survival analysis approach Traffic Injury Prevention, 22(2), 153–157. 10.1080/15389588.2020.1853711. 33337927

[50] Kaplan EL, Meier P, (1958). Nonparametric estimation from incomplete observations. Journal of the American Statistical Association, 53(282), 457–481. 10.2307/2281868.

[51] Liu QY, Sun J, Tian Y, Ni Y, Yu SA, (2019). Modeling and simulation of nonmotorized vehicles’ dispersion at mixed flow intersections. Journal of Advanced Transportation, 2(2019), 1–19. 10.1155/2019/9127062.

[52] Cameron C, Trivedi PK. Microeconometrics: Methods and APP lieations [M]. New York: Cambridge University Press, 2005: 573–600.

[53] Zarova E, Зароbа ЕВ, (2012). International encyclopedia of statistical science-significant contribution into the future of statistics. Voprosy Statistiki, 5, 82–85.

